# Risk of Suicidal Behaviors and Antidepressant Exposure Among Children and Adolescents: A Meta-Analysis of Observational Studies

**DOI:** 10.3389/fpsyt.2022.880496

**Published:** 2022-05-26

**Authors:** Kuan Li, Guibao Zhou, Yan Xiao, Jiayu Gu, Qiuling Chen, Shouxia Xie, Junyan Wu

**Affiliations:** ^1^Department of Pharmacy, Shenzhen People’s Hospital, The Second Clinical Medical College, Jinan University, The First Affiliated Hospital, Southern University of Science and Technology, Shenzhen, China; ^2^Shenzhen Key Laboratory of Prevention and Treatment of Severe Infections, Shenzhen People’s Hospital, The Second Clinical Medical College, Jinan University, The First Affiliated Hospital, Southern University of Science and Technology, Shenzhen, China; ^3^Department of Pharmacy, Sun Yat-sen Memorial Hospital, Sun Yat-sen University, Guangzhou, China

**Keywords:** antidepressants, SSRIs, suicide, suicide attempt, children and adolescents

## Abstract

**Objectives:**

Although several studies have reviewed the suicidal risk of antidepressants, the conclusions remain inconsistent. We, therefore, performed a meta-analysis of observational studies to address the association between exposure to antidepressants, especially selective serotonin reuptake inhibitors (SSRIs) and the risk of suicide and suicide attempt in children and adolescents.

**Methods:**

MEDLINE and Embase were searched from January 1990 to April 2021. Seventeen cohort and case-control studies were identified that reported suicide or suicide attempt in children and young adults (aged 5–25 years) who were exposed to any antidepressants. We extracted the estimates and corresponding 95% confidence intervals (CIs) from each publication.

**Results:**

The results showed that antidepressant exposure significantly increased the risk of suicide and suicide attempt when compared with no antidepressant usage among children and adolescents. The pooled relative risk (RR) was 1.38 (95% CI: 1.16–1.64; *I*^2^ = 83.1%). Among the antidepressants, SSRI use was associated with an increased risk of suicide and suicide attempt, and the pooled RR was 1.28 (95% CI: 1.09–1.51; *I*^2^ = 68.8%). In subgroup analysis, the attempted suicidal risk of antidepressant and SSRI was significantly increased (RR = 1.35, 95% CI: 1.13–1.61; *I*^2^ = 86.2% for all antidepressants; and RR = 1.26, 95% CI: 1.06–1.48; *I*^2^ = 73.8% for SSRIs), while the completed suicidal risk of antidepressant and SSRI was not statistically significant (RR = 2.32, 95% CI: 0.82–6.53; *I*^2^ = 6.28% for all antidepressants; and RR = 1.88, 95% CI: 0.74–4.79; *I*^2^ = 52.0% for SSRIs). In addition, the risk of suicide and suicide attempt between SSRIs and other antidepressants was similar (RR 1.13, 95% CI: 0.87–1.46, *I*^2^ = 32.4%).

**Conclusion:**

The main findings of this meta-analysis provide some evidence that antidepressant exposure seems to have an increased suicidal risk among children and young adults. Since untreated depression remains one of the largest risk factors for suicide and the efficacy of antidepressants is proven, clinicians should evaluate carefully their patients and be cautious with patients at risk to have treatment emergence or worsening of suicidal ideation (TESI/TWOSI) when prescribing antidepressants to children and young patients.

## Introduction

Accumulating evidence suggests that antidepressant use in children and adolescents is substantially increased in recent years (1), though the FDA issued a “black box” warning for selective serotonin reuptake inhibitors (SSRIs) of suicidal thinking and behavior in children and adolescents in 2004 (2) and expanded to include young adults (aged 18–24 years) in 2007 (3). SSRIs are the most commonly prescribed antidepressants for the treatment of depressive and anxiety disorders (1) and the relationship between SSRIs and other antidepressants, and the risk of suicidal behavior (including completed suicide and suicide attempt) has been subject to considerable public attention since 2004, especially in young people. Likewise, clinical guidelines covering the use of antidepressants in children and adolescents have taken the suicidal risk into consideration and recommended careful monitoring for suicidal behaviors after initiation of antidepressant treatment (4). Suicide is one of the major causes of death with a rate of 534.3 per 100,000 person-years in patients with major depression (5), and suicide clusters occur more frequently in young people than in adults, especially through social media (6, 7); thus, it is of great importance to identify the suicidal risk of antidepressants.

Suicidal ideation and behavior developed during depression treatment are called treatment emergence or worsening of suicidal ideation (TESI/TWOSI). The incidence of TESI varies from 3.2 to 17% among different studies, depending on the studied population and the threshold used for TESI (8). Predictors associated with TESI and TWOSI include sociodemographic and clinical risk factors (i.e., the preadult onset of depression, gender, depression severity, physical pain, and poor response to antidepressants) (8, 9) and also genetic risk factors (10, 11). Also, effective temperament types were independently and strongly associated with lifetime suicide attempts (12). However, whether antidepressants would increase the suicidal ideation and behavior in children and adolescents remain controversial, and an updated review of new evidence is needed as the prevalence of antidepressant use in children and adolescents is growing in the last decade (13–15), for example, the prevalence of antidepressant use in children and adolescents increased from 13 to 16% in the USA and from 07 to 11% in the United Kingdom in 2005–2012 (15).

Meta-analysis of the randomized controlled trials (RCTs), the highest level of evidence, produced inconsistent findings. Unlike the meta-analysis (16) conducted a decade ago, which found that the overall risk ratio for SSRIs in depressed pediatric patients was 1.66 (95% CI: 1.02–2.68), the recent meta-analysis of RCTs (17, 18) gives the conclusion that only venlafaxine was found to be associated with an increased risk of suicidal behavior or ideation in the young population, and the suicidal risk of other antidepressants remains unclear due to the absence of reliable data. As most trials on antidepressants excluded patients with suicidal ideation and behavior (19, 20), RCTs only provided limited assessments of antidepressant use on suicidal risk, leading to uncertainty about medication safety and efficacy in this specific population. However, it is possible to overcome the barriers to include suicidal participants and conduct the clinical trials safely, as seen with recent research on esketamine for example (9, 21), and inclusion of this specific population into psychiatric clinical trials in the future is critical to efforts to reduce suicidal rates. In addition, observational studies, which were conducted in the real world and included a large, broad spectrum of individuals with long duration, might also offer a reliable suggestion for clinical services and could be complementary to that provided by clinical trials.

A recent umbrella review (22) of observational studies evaluated the adverse outcomes of antidepressants, and the only convincing evidence found is the association between antidepressant use and the risk of suicide attempt or completion among children and adolescents, which was published in 2009 (23). Many studies were published since then, which might affect the conclusion. Therefore, we conducted an updated meta-analysis of observational studies to further address the association between antidepressant exposure and the risk of suicide and suicide attempt in children and adolescents.

## Methods

### Search Strategy

We followed the checklist of Preferred Reporting Items for Systematic Reviews and Meta-Analyses (PRISMA) for background, design, analysis, and interpretation. We conducted a literature search of MEDLINE/PubMed, Embase, and Cochrane Library from January 1990 to April 2021 for relevant studies assessing the association between antidepressants and the risk of suicide. Various combinations of keywords were utilized, including but not limited to (“antidepressant,” “antidepressive,” and “SSRI”) and (“suicide,” “suicide attempt,” and “suicidal behavior”). We also scrutinized the reference lists of relevant major reviews. No language restrictions were imposed. The detailed search strategy and the PubMed search term are shown in Supplementary Table 1.

### Study Selection

Eligible studies were included in this meta-analysis if they satisfied the following criteria: (1) the study design was observational cohort or case-control studies, (2) the study population include children or young patients (age < 25 years), (3) the exposure of interest was antidepressant use, and (4) the outcomes were suicidal behaviors (i.e., completed suicide or suicide attempt) or deliberate self-harm (with a clear definition to include suicidal behavior). One reviewer assessed the titles and abstracts of all studies identified through electronic searches. Potentially eligible studies were reviewed independently by a second reviewer, with discrepancies resolved by discussion.

### Data Extraction

Two reviewers (ZGB and XY) independently extracted and collected the data. The following information was extracted from each eligible study: name of the first author, year of publication, study design, study location, mean follow-up time, participants’ characteristics, number of participants, outcomes, outcome assessment methods, and adjustment for potential confounders. We extracted crude and adjusted estimates and corresponding 95% confidence intervals (CIs). When studies had several adjustment models, we used the most comprehensively adjusted estimates in multivariable-adjusted models. Crude relative risks (RRs) or odds ratios (ORs) were calculated when the case numbers in the exposure group and comparison group were shown but the risk estimates remain unknown.

Quality assessment was performed using the Newcastle-Ottawa Scale (NOS) (24). The maximum NOS score for an observational study is 9 points (4 points for selection, 2 points for comparability, and 3 points for outcome).

### Statistical Analysis

Since the incidence of suicide and suicide attempt was low, we regarded OR as close approximations of RR and combined them with a hazard ratio (HR), resulting in a common estimate of RR. Potential heterogeneity among studies was estimated using the Cochran Q test and *I*^2^ statistic (25). *I*^2^ can be interpreted as the proportion of the total variance due to heterogeneity. Where the *I*^2^ estimate was greater than or equal to 50%, we interpreted this as indicating the presence of high levels of heterogeneity. We used a fixed-effect model (Mantel-Haenszel method) when heterogeneity was negligible, and a random effect model (DerSimonian and Laird method) when heterogeneity was significant (26). In addition, we used restricted maximum likelihood random-effects meta-regression to explore heterogeneity by the publication year of study, length of follow-up, the outcome, and the diagnosis of depression. The possibility of publication bias was evaluated using the Egger regression asymmetry test (27) and the Begg test. Sensitivity analyses for the influence of single studies on the pooled RR were conducted by omitting studies one by one and reestimating the pooled RR. All statistical analyses were performed using Stata version 12 (Stata Corp).

## Results

### Study Selection and Characteristics

**FIGURE 1:**
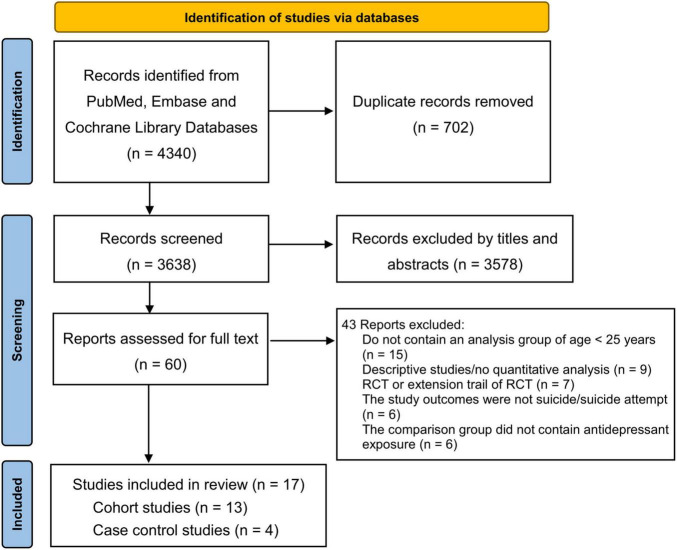
Flow diagram of literature search and study selection.

Figure 1 shows a flow diagram for the study selection process, the literature search identified 4,340 references that included antidepressant exposure and suicide or suicide attempt, which were reduced to 3,638 after removal of duplicates, and 3,578 references were excluded as not being relevant by review of the title and abstract. After evaluating the full texts of these 60 publications, 43 studies were excluded (Supplementary Table 2), and 17 independent studies (28–44) meeting the inclusion criteria were assessed in this meta-analysis.

The characteristics of 17 identified studies are shown in Supplementary Table 3, 10 studies (28, 30, 33, 34, 38–40, 42–44) were conducted in the United States and seven studies (29, 31, 32, 35–37) in Europe. Of the included studies, 13 were cohort studies and 4 were case-control studies. Overall follow-up time ranged from 4 months to 17.7 years, with a weighted mean of 2.1 years. Two studies reported estimates on more than one outcome (30, 36) (suicide and suicide attempt), and fifteen studies reported only one outcome (suicide or suicide attempt). Fourteen studies used ICD-9 or ICD-10 codes to define suicide and suicide attempt and the other three used medical records. One study reported the results of two databases separately (40). The results of the study quality assessment (score 0–9) yielded scores of 6 or above (high quality) for fifteen studies and 5 for two studies, with an average score of 7.1.

### Antidepressant Exposure and the Risk of Suicide and Suicide Attempt Among Adolescents

**FIGURE 2:**
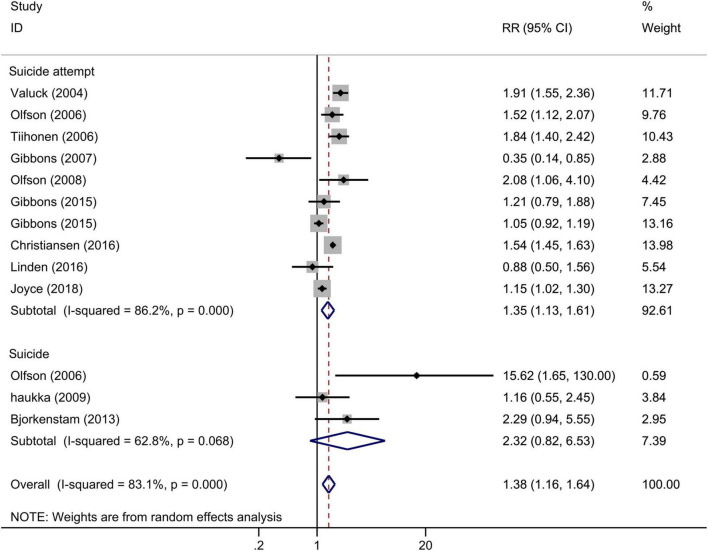
Forest plot of antidepressant exposure and the risk of suicidal behavior among children and adolescents.

Among 11 studies that examined the association between any antidepressants (including SSRIs) use and completed or attempted suicide among adolescents (13 estimates), antidepressant exposure significantly increased the risk of completed or attempted suicide when compared with no antidepressant exposure (Figure 2), and the overall RR for incidence of suicide or suicide attempt was 1.38 (95% CI: 1.16–1.64, *I*^2^ = 83.1%, *P* < 0.001). To explore the potential source of heterogeneity across studies, a sensitivity analysis was performed by removing one study at a time, and the corresponding pooled RR was not materially altered after the removal of any study (Supplementary Figure 1). We performed a meta-regression with publication year and length of follow-up as covariates in the meta-analysis. The results showed that the publication year of the study explains 50.98% of heterogeneity (*P* = 0.026). Neither the Begg test nor the Egger regression test for publication bias reached significance (*P* > 0.05 for both tests).

In the subgroup analysis according to the outcomes (Figure 2), antidepressant exposure significantly increased the risk of suicide attempt (RR = 1.35, 95% CI: 1.13–1.61, *I*^2^ = 86.2%, *P* < 0.001), with no significant effect on completed suicide (RR = 2.32, 95% CI: 0.82–6.53, *I*^2^ = 62.8%, *P* = 0.068). We also performed subgroup analyses according to the adjustment, and the pooled adjusted and crude RR showed similar results (Supplementary Figure 2). The result of pooled adjusted estimate suggested that the results of the overall meta-analysis were robust.

### Selective Serotonin Reuptake Inhibitors Exposure and the Risk of Suicide and Suicide Attempt Among Adolescents

**FIGURE 3:**
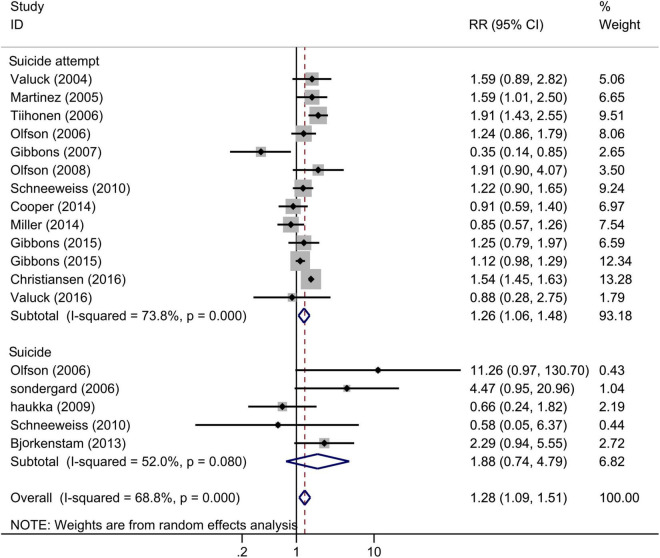
Forest plot of selective serotonin reuptake inhibitor (SSRI) exposure and the risk of suicidal behavior among children and adolescents.

Across 15 studies that examined the association between SSRIs and completed or attempted suicide among adolescents (18 estimates), SSRI exposure significantly increased the risk of completed and attempted suicide (Figure 3), and the pooled RR for incidence of suicide or suicide attempt was 1.28 (95% CI: 1.09–1.51, *I*^2^ = 68.8%, *P* < 0.001). The control group was no antidepressant use or any other antidepressant use. This result was robust in the sensitivity analysis (Supplementary Figure 3). Results from separate meta-regression models suggested that the publication year (*P* > 0.05) and follow-up duration (*P* > 0.05) were not the sources of heterogeneity. We found no evidence of publication bias (*P* > 0.05 for Begg and Egger tests).

In the subgroup analysis according to the outcomes (Figure 3), SSRI exposure significantly increased the risk of suicide attempt (RR = 1.26, 95% CI: 1.06–1.48, *I*^2^ = 73.8%, *P* < 0.001), with no significant effect on completed suicide (RR = 1.88, 95% CI: 0.74–4.79, *I*^2^ = 52.0%, *P* = 0.08). In the subgroup analysis of adjustment (Supplementary Figure 4), the pooled adjusted RR was 1.42 (95% CI 1.18–1.72; *I*^2^ = 69.7%), similar to analysis that included all studies, while the pooled crude RR was 1.11 (95% CI 0.83–1.50; *I*^2^ = 55.1%).

**FIGURE 4:**
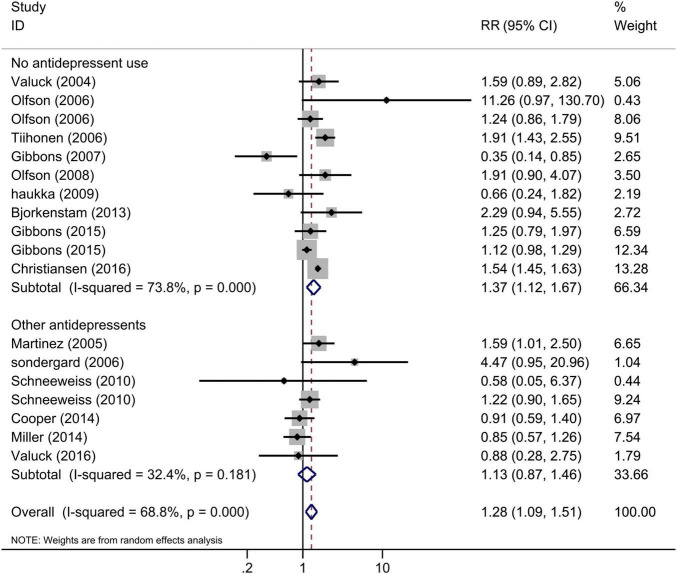
Subgroup analysis of SSRI exposure and risk of suicidal behavior according to the comparisons.

The comparisons of antidepressant exposure were no antidepressant exposure in all included studies, and the comparisons of SSRI exposure were any other antidepressant use or no antidepressant use. Thus, we performed the subgroup analysis based on the comparisons, and the results indicated that the risk of suicide/suicide attempt of SSRI use was significantly increased compared with no antidepressant use (Figure 4, RR = 1.37, 95% CI: 1.12–1.67, *I*^2^ = 73.8%, *P* < 0.001), but similar to other antidepressants (Figure 4, RR = 1.13, 95% CI: 0.87–1.46, *I*^2^ = 32.4%, *P* = 0.18). This subgroup analysis indicated that the increased risk of suicidal behavior for SSRIs and other antidepressants was similar among children and adolescents. Since the subgroup finding was based on a small number of studies, the results should be interpreted with caution.

## Discussion

Our meta-analysis identified 17 observational studies and provided a comprehensive review and quantitative estimates of the association between antidepressant exposure and the risk of suicide and suicide attempt. The results showed that antidepressant use in children and young populations increased the risk of suicidal behavior compared with no antidepressant use. Especially, the exposure to only SSRIs also increased the risk of the suicide attempt. These results were consistent with the main conclusion of the previous review of clinical trial data (23, 45, 46). Similar results were obtained in subgroup analyses of a suicide attempt; however, the increased risk of completed suicide exposed to antidepressants was not significant among adolescents, suggesting that the risk of completed suicide might be discordant with attempted suicide. Together, these results suggested that adolescents and young adults might be at increased risk for suicidal behaviors following antidepressant therapy, including SSRIs.

A few studies have investigated clinical and genetic risk factors of TESI/TWOSI in adults that can contribute to the increased risk of suicidal behavior at the antidepressant onset. The results showed that the severity of depression, the first few weeks of treatment, drug abuse, poor response to antidepressants, physical pain, and previous history of suicidal behavior or ideation was associated with the emergence of TESI/TWOSI (41, 47–51). In addition, some of the few available studies about genetic risk factors of TESI/TWOSI reported associations with single nucleotide polymorphisms of genes involved in the neurotropic and synaptic plasticity systems (52, 53), noradrenergic system (52), glutamatergic system (54), stress and inflammatory responses (11, 55), and opioid system (56). Those clinical and genetic risk factors reported in adults may also contribute to the TESI/TWOSI in children and adolescents and, thus, help to monitor the TESI/TWOSI during treatment.

The mechanisms of the increased suicidal risk of antidepressants among adolescents were not quite clear; however, some studies in animal models have found that treatment with SSRIs could exert potent anxiogenic behavioral effects, in particular during the acute phase of treatment (57–59). The inhibitory circuit of corticotropin-releasing factor neurons in the bed nucleus of the stria terminalis may contribute to the aversive behavior following acute exposure to SSRIs (58). Furthermore, another research (60) on an animal model reported that brief administration of paroxetine in young rats can promote, rather than reduce, the depressive state, contrary to the therapeutic changes observed in adult rats. Those findings might partially explain the adverse effects of antidepressants in adolescents.

Recently, a few studies reported that intranasal esketamine or ketamine may result in significantly rapid improvement in depressive symptoms and suicidal ideation among depressed patients at imminent risk for suicide (21, 61), and as previous history of suicidal behavior or ideation and poor response to antidepressants are both risk factors for TESI/TWOSI, treatment with esketamine or ketamine may be an option to be further explored among children and adolescents with those risk factors. In addition, dialectical behavior therapy is reported to have beneficial effects on self-harm in children and adolescents, and still, further evaluation for dialectical behavior therapy and cognitive behavior therapy is warranted (62).

### Comparable With Other Studies

In line with our results, several RCTs found a significant increase in the incidence of suicide attempts for adolescents and young adults receiving antidepressants compared with placebo (63, 64), including the analyses conducted by the FDA (16, 65). Bridge (66) analyzed 27 RCTs of second-generation antidepressants in participants younger than 19 years with major depressive disorder, obsessive-compulsive disorder (OCD), or non-OCD anxiety disorders and found an overall increased risk of suicidal ideation/suicide attempt associated with antidepressant treatment. Also, Hetrick et al. (45) performed a systematic review of published and unpublished randomized controlled trials comparing newer generation antidepressants (mainly SSRIs) with placebo in children and adolescents aged 6–18 years. Statistical analysis revealed an increased risk (58%) of suicide-related outcomes for those on antidepressants compared with placebo (RR = 1.58, 95% CI: 1.02–2.45). Moreover, our result among adolescents was similar to the previous systemic review (23) of observational studies which found that exposure to SSRIs almost doubled the risk of suicide among adolescents.

Counter to the evidence showing an increase in suicide-related behaviors, some arguments have been mounted. Several recent meta-analyses of RCTs (17, 18, 67) found no statistically significant risk between suicide-related outcomes and antidepressant use, except venlafaxine. But children and adolescents considered at risk of suicide were frequently excluded from RCTs so the proportions of suicide-related outcomes in those trials were low for most included studies and 95% CIs were wide for all comparisons. Otherwise, a review (68) aggregated data from six population studies of adolescent suicides that contained individual data on SSRIs at or around the time of death, and the results found no evidence that SSRIs were associated with increased suicide in young people. Wijlaars (69) performed a self-control study and revealed no systematic differences between the association of SSRIs and the incidence risk ratios for attempted suicide or intentional self-harm. But some important confounders such as depression severity were not controlled in both studies. In addition, numerous population-based studies (70–72) have shown an inverse association between antidepressant use and suicide rates. Another study (73) found suicide in the 10–19-year age group increased for five consecutive years (60.5%), and the increase occurred among individuals not treated with antidepressants. However, this negative association does not allow conclusion to be drawn regarding causality, and it is crucial to control for potential confounding factors. Consequently, the balance between risks and benefits will need to be considered for individual patients.

It is unclear whether the risk for antidepressant use is discrepant between completed suicide and attempted suicide. Consistent with our subgroup analysis, a meta-review (74) of a systematic review study found that SSRIs may increase suicidal thoughts, but not actual suicide in early phase therapy. Tiihonen (32) suggested that antidepressant use was associated with an increased risk of non-fatal suicidal behavior and a decreased risk of fatal suicidal behavior. The possible explanations for this finding include that: (i) the subgroup analysis on completed suicide was based on a small number of studies (three for antidepressants and five for SSRIs, respectively); (ii) the number of completed suicidal cases is limited in most included studies. Thus, this result that the increased risk of completed suicide exposed to antidepressants was not significant among adolescents should be interpreted with caution.

### Strength and Limitations

Our analysis has several strengths. We did sensitivity analyses for the influence of single studies on the pooled RR which suggested that no individual study had excessive influences on the main results. We pooled adjusted estimates in the subgroup analysis, using models adjusting for most established risk factors. Also, most studies adjusted for potential risk for suicide such as age, sex, and psychiatric characteristics (e.g., psychiatric contact or hospitalization, history of suicidal behavior, severity of depression, and multiple antidepressant medications), and the adjustments of each study are listed in Supplementary Table 3. Additionally, almost all studies used ICD-9 or ICD-10 codes to define suicide and suicide attempt, making sure that the standards of the outcomes stayed consistent. However, we found a great level of heterogeneity, and this may be due to the publication year of the study according to the meta-regression since the estimate of suicidal risk would be affected after the “black box” warning issued by FDA.

Several limitations of our study should also be acknowledged. First, a time-dependent decline in the suicidal rate for antidepressant recipients was identified (75); however, we did not analyze the association between prescription time and suicidal risk owing to the absence of reliable data. Second, a meta-analysis of observational data has limited ability to adjust for baseline differences and specific risk factors and is prone to bias and confounding. We made an attempt to allow for multiple confounding by including adjusted estimates from multivariate models in each contributing study. However, due to limited data from the original studies, we were unable to stratify studies by sex, severity of illness, species of SSRIs, or the history of the suicide attempt. Third, not all studies made enough adjustments for confounders. For example, some risk factors for suicidal behavior (e.g., personality disorder, physical disease, and childhood irritability) are not adjusted in the included studies (76–78), also, not all the studies adjusted their results on lifetime history of suicide attempts and other suicidal behavior characteristics. Thus, results from the main analyses were affected by various confounders. It is important that future studies specify those confounding factors. Fourth, no inference can be made about newer antidepressants (e.g., vortioxetine hydrobromide) that have not been assessed in any of the included analyses. Fifth, although we carried out several subgroup analyses and meta-regression, the substantial heterogeneity present in most analyses remains unexplained. Finally, observational studies cannot provide causal evidence of the effect of antidepressant use on suicidal behaviors; they can describe only associations.

## Conclusion

The main findings of this meta-analysis provide some evidence that antidepressant exposure seems to have an increased suicidal risk among children and young adults. Since untreated depression remains one of the largest risk factors for suicide and the efficacy of antidepressants is proven, clinicians should evaluate carefully their patients and be cautious with patients at risk to have TESI and TWOSI when prescribing antidepressants to children and young patients (8). The most advantageous treatment or combination of non-pharmacological interventions should be considered for this specific population. Moreover, esketamine and ketamine are reported to transiently decrease suicidal ideation in patients with serious suicidal thoughts or actions, yet the long-term outcomes and safety in children and young adults are still not clear (79). Given the potential for life-threatening events in young children and adolescents, it is essential to seek and evaluate new strategies for young patients at risk.

## Data Availability Statement

The original contributions presented in the study are included in the article/Supplementary Material, further inquiries can be directed to the corresponding authors.

## Author Contributions

KL and JW conceived and designed this study. JG and QC conducted the databases search. GZ and YX selected the studies, extracted the data, and assessed the risk of bias. KL was in charge of writing-reviewing of the manuscript. SX and JW took supervision. All authors contributed to this study and approved the final manuscript.

## Conflict of Interest

The authors declare that the research was conducted in the absence of any commercial or financial relationships that could be construed as a potential conflict of interest.

## Publisher’s Note

All claims expressed in this article are solely those of the authors and do not necessarily represent those of their affiliated organizations, or those of the publisher, the editors and the reviewers. Any product that may be evaluated in this article, or claim that may be made by its manufacturer, is not guaranteed or endorsed by the publisher.
