# Supplementary material for: Risk of Suicidal Behaviors and Antidepressant Exposure Among Children and Adolescents: A Meta-Analysis of Observational Studies
Source: Front Psychiatry. 2022 May 26;13:880496. doi: 10.3389/fpsyt.2022.880496 (PMC9178080; doi:10.3389/fpsyt.2022.880496)
Supplement: Supplementary file 1 [file Data_Sheet_1.docx]

# Supplementary Tables

**Supplementary table 1. Pubmed search strategy**

| #1 | Serotonin Uptake Inhibitors[Mesh] OR 5-Hydroxytryptamine Uptake Inhibitors[tiab] OR 5 Hydroxytryptamine Uptake Inhibitors[tiab] OR inhibitors, 5-HT Uptake[tiab] OR Inhibitors, 5 HT Uptake[tiab] OR Inhibitors, 5-Hydroxytryptamine Uptake[tiab] OR Inhibitors, 5 Hydroxytryptamine Uptake[tiab] OR Inhibitors, Serotonin Reuptake[tiab] OR Reuptake Inhibitors, Serotonin[tiab] OR Serotonin Reuptake Inhibitors[tiab] OR Uptake Inhibitors, 5-HT[tiab] OR Uptake Inhibitors, 5 HT[tiab] OR Uptake Inhibitors, 5-Hydroxytryptamine[tiab] OR Uptake Inhibitors, 5 Hydroxytryptamine[tiab] OR Uptake Inhibitors, Serotonin[tiab] OR 5-HT Uptake Inhibitors[tiab] OR 5 HT Uptake Inhibitors[tiab] OR Inhibitors, Serotonin Uptake[tiab] OR Selective Serotonin Reuptake Inhibitors[tiab] OR SSRI*[tiab] OR Antidepressant Drugs[tiab] OR Antidepressants[Mesh] OR Serotonin and Noradrenaline Reuptake Inhibitors[Mesh] or serotonin norepinephrine reuptake inhibitor*[tiab] or SNRI[tiab] OR citalopram[Mesh] or fluoxetine[Mesh] or paroxetine[Mesh] or sertraline[Mesh] or fluvoxamine[Mesh] or venlafaxine[Mesh] or duloxetine[Mesh] or milnacipran[Mesh] or reboxetine[Mesh] or bupropion[Mesh] or mirtazapine[Mesh] or Antidepressive Agents, Tricyclic[Mesh] or amitriptyline[Mesh] or amoxapine[Mesh] or clomipramine[Mesh] or desipramine[Mesh] or dothiepin[Mesh] or doxepin[Mesh] or imipramine[Mesh] or lofepramine[Mesh] or nortriptyline[Mesh] or opipramol[Mesh] or protriptyline[Mesh] or trimipramine[Mesh] or citalopram[tiab] or fluoxetine[tiab] or paroxetine[tiab] or sertraline[tiab] or escitalopram[tiab] or fluvoxamine[tiab] or venlafaxine[tiab] or duloxetine[tiab] or milnacipran[tiab] or reboxetine[tiab] or bupropion[tiab] or noradrenergic[tiab] OR specific serotonergic antidepressants[tiab] or NaSSA[tiab] or mirtazapine[tiab] or TCA[tiab] or tricyclic[tiab] or amersergide[tiab] or amineptine[tiab] or amitriptyline[tiab] or amoxapine[tiab] or butriptyline[tiab] or clomipramine[tiab] or clorimipramine[tiab] or demexiptiline[tiab] or desipramine[tiab] or dothiepin[tiab] or doxepin[tiab] or imipramine[tiab] or lofepramine[tiab] or melitracen[tiab] or metapramine[tiab] or nortriptyline[tiab] or noxiptiline[tiab] or opipramol[tiab] or protriptyline[tiab] or quinupramine[tiab] or tianeptine[tiab] or trimipramine[tiab] |
| --- | --- |
| #2 | child[Mesh] or adolescent[Mesh] or minors[Mesh] or pediatrics[Mesh] or young adult[Mesh] OR adolesc*[tiab] or child*[tiab] or boy*[tiab] or girl*[tiab] or juvenil*[tiab] or minors[tiab] or paediatri*[tiab] or pediatri*[tiab] or pubescen*[tiab] or school*[tiab] or student*[tiab] or teen*[tiab] or young[tiab] or youth*[tiab] |
| #3 | (("suicide"[MeSH Terms] OR "suicide"[All Fields]) OR ("suicidal ideation"[MeSH Terms] OR ("suicidal"[All Fields] AND "ideation"[All Fields]) OR "suicidal ideation"[All Fields]) OR (("suicide"[MeSH Terms] OR "suicide"[All Fields]) |
| #4 | #1 AND #2 AND #3 |

**Supplementary table 2.** **Articles Excluded After Full-Text Revision With Reasons**

| 1 | Anderson, H. D., et al. (2012). "Rates of 5 common antidepressant side effects among new adult and adolescent cases of depression: a retrospective US claims study." Clinical Therapeutics 34(1): 113-123. | The study outcomes were not suicide/suicide attempt/self-harm |
| --- | --- | --- |
| 2 | Atkinson, S., et al. (2018). "Desvenlafaxine Versus Placebo in the Treatment of Children and Adolescents with Major Depressive Disorder." Journal of Child and Adolescent Psychopharmacology 28(1): 55-65. | RCT |
| 3 | Beckman, K., et al. (2016). "Mental illness and suicide after self-harm among young adults: long-term follow-up of self-harm patients, admitted to hospital care, in a national cohort." Psychological Medicine 46(16): 3397-3405. | The comparison group did not contain antidepressant exposure |
| 4 | Bhoombla, N., et al. (2020). "Pharmacovigilance Reports Received from Children and Young People, and Development of Information to Aid Future Reporting from this Age Group." Paediatric Drugs 22(3): 335-341. | The comparison group did not contain antidepressant exposure |
| 5 | Cairns, R., et al. (2019). "Trends in self-poisoning and psychotropic drug use in people aged 5-19 years: a population-based retrospective cohort study in Australia." BMJ Open 9(2): e026001. | no quantitative analysis of the association of antidepressant use with self-poisoning |
| 6 | Castelpietra, G., et al. (2016). "Diagnoses and prescriptions of antidepressants in suicides: Register findings from the Friuli Venezia Giulia Region, Italy, 2002-2008." International Journal of Psychiatry in Clinical Practice 20(2): 121-124. | do not contein a analysis group of age < 24 years. case control |
| 7 | Castelpietra, G., et al. (2017). "Antidepressant use in suicides: a case-control study from the Friuli Venezia Giulia Region, Italy, 2005-2014." European Journal of Clinical Pharmacology 73(7): 883-890. | do not contein a analysis group of age < 24 years. |
| 8 | Cheung, K., et al. (2015). "Antidepressant use and the risk of suicide: a population-based cohort study." Journal of Affective Disorders 174: 479-484. | do not contein a analysis group of age < 24 years cohort |
| 9 | Coupland, C., et al. (2015). “Antidepressant use and risk of suicide and attempted suicide or self harm in people aged 20 to 64: cohort study using a primary care database.” BMJ 350: h517 | cohort do not contein the group of age < 24 years. |
| 10 | Coupland, C., et al. (2018). "Antidepressant use and risk of adverse outcomes in people aged 20-64 years: cohort study using a primary care database." BMC Medicine 16(1): 36. | The study outcomes were not suicide/suicide attempt |
| 11 | Cousins, L., et al. (2016). "Clinical characteristics associated with the prescribing of SSRI medication in adolescents with major unipolar depression." European Child and Adolescent Psychiatry 25(12): 1287-1295. | The study outcomes were not suicide/suicide attempt |
| 12 | Didham, R. C., et al. (2005). "Suicide and self-harm following prescription of SSRIs and other antidepressants: confounding by indication." British Journal of Clinical Pharmacology 60(5): 519-525. | cohort do not contein the group of age < 24 years. |
| 13 | Eikelenboom, M., et al. (2019). "A 6-year longitudinal study of predictors for suicide attempts in major depressive disorder." Psychological Medicine 49(6): 911-921. | do not contein a analysis group of age < 24 years. |
| 14 | Emslie, G. J., et al. (2015). "Acute and longer-term safety results from a pooled analysis of duloxetine studies for the treatment of children and adolescents with major depressive disorder." Journal of Child and Adolescent Psychopharmacology 25(4): 293-305. | pool analysis of RCT |
| 15 | Erlangsen, A., et al. (2009). "Early discontinuation of antidepressant treatment and suicide risk among persons aged 50 and over: a population-based register study." Journal of Affective Disorders 119(1-3): 194-199. | do not contein the group of age < 24 years |
| 16 | Findling, R. L., et al. (2013). "Escitalopram in the treatment of adolescent depression: a randomized, double-blind, placebo-controlled extension trial." Journal of Child and Adolescent Psychopharmacology 23(7): 468-480. | extension trail of RCT |
| 17 | Fontanella, C. A., et al. (2009). "Psychotropic medication changes, polypharmacy, and the risk of early readmission in suicidal adolescent inpatients." Annals of Pharmacotherapy 43(12): 1939-1947. | The study outcomes were not suicide/suicide attempt/self-harm |
| 18 | Forsman, J., et al. (2019). "Selective serotonin re-uptake inhibitors and the risk of violent suicide: a nationwide postmortem study." European Journal of Clinical Pharmacology 75(3): 393-400. | do not contein a analysis group of age < 24 years. |
| 19 | Froberg, B. A., et al. (2019). "Temporal and geospatial trends of adolescent intentional overdoses with suspected suicidal intent reported to a state poison control center." Clinical Toxicology (Philadelphia, Pa.) 57(9): 798-805. | no quantitative analysis of the association of antidepressant use with suicide |
| 20 | Ghaziuddin, N., et al. (2014). "A naturalistic study of suicidal adolescents treated with an SSRI: suicidal ideation and behavior during 3-month post-hospitalization period." Asian Journal of Psychiatry 11: 13-19. | no quantitative analysis of the association of antidepressant use with suicide |
| 21 | Hartz, I., et al. (2016). "Antidepressant drug use among adolescents during 2004-2013: a population-based register linkage study." Acta Psychiatrica Scandinavica 134(5): 420-429. | The study outcomes were not suicide/suicide attempt |
| 22 | Hogberg, G., et al. (2015). "Suicidal risk from TADS study was higher than it first appeared." International Journal of Risk & Safety in Medicine 27(2): 85-91. | pool analysis of RCT |
| 23 | Jick, H., et al. (2004). "Antidepressants and the risk of suicidal behaviors." JAMA 292(3): 338-343. | do not contein the group of age < 24 years. |
| 24 | Miller, M., et al. (2014). "Antidepressant dose, age, and the risk of deliberate self-harm." JAMA Intern Med 174(6): 899-909. | high dose vs modal-dose The comparison group did not contain non-antidepressant exposure |
| 25 | Mosheva, M., et al. (2016). "Do Antidepressants Induce Psychosis in Children and Adolescents? A Naturalistic Study in Ambulatory Pediatric Population." Journal of Child and Adolescent Psychopharmacology 26(5): 478-484. | The comparison group did not contain antidepressant exposure |
| 26 | Ogino, Y. and A. J. Schmidt (2020). "Impact of class-level labelling change on prescriptions of antidepressants for adolescents: An interrupted time-series study using a health insurance claims database in Japan, 2005-2013." PloS One 15(12): e0243424. | The comparison group did not contain antidepressant exposure |
| 27 | Olmer, A., et al. (2012). "Exposure to antidepressant medications and suicide attempts in adult depressed inpatients." Journal of Nervous and Mental Disease 200(6): 531-534. | do not contein the group of age < 24 years |
| 28 | Overberg, A., et al. (2019). "Toxicity of Bupropion Overdose Compared With Selective Serotonin Reuptake Inhibitors." Pediatrics 144(2). | no quantitative analysis of the association of antidepressant use with suicide |
| 29 | Rynn, M. A., et al. (2015). "Child/Adolescent anxiety multimodal study: evaluating safety." Journal of the American Academy of Child and Adolescent Psychiatry 54(3): 180-190. | reanalysis of RCT |
| 30 | Schneeweiss, S., et al. (2010). "Variation in the risk of suicide attempts and completed suicides by antidepressant agent in adults: a propensity score-adjusted analysis of 9 years' data." Archives of General Psychiatry 67(5): 497-506. | do not contein the group of age < 24 years. cohort |
| 31 | Seemüller, F., et al. (2009). "The controversial link between antidepressants and suicidality risks in adults: data from a naturalistic study on a large sample of in-patients with a major depressive episode." International Journal of Neuropsychopharmacology 12(2): 181-189. | no quantitative analysis of the association of antidepressant use with suicide |
| 32 | Sheridan, D. C., et al. (2018). "Suicidal bupropion ingestions in adolescents: increased morbidity compared with other antidepressants." Clinical Toxicology (Philadelphia, Pa.) 56(5): 360-364. | no quantitative analysis of the association of antidepressant use with suicide |
| 33 | Sondergard, L., et al. (2007). "Continued antidepressant treatment and suicide in patients with depressive disorder." Arch Suicide Res 11(2): 163-175. | do not contein the group of age < 24 years. |
| 34 | Sorensen, J. O., et al. (2020). "Clinician compliance to recommendations regarding the risk of suicidality with selective serotonin reuptake inhibitors in the treatment of children and adolescents." European Child and Adolescent Psychiatry 29(5): 707-718. | The comparison group did not contain antidepressant exposure |
| 35 | Spittal, M. J., et al. (2019). "Modifiable risk factors for external cause mortality after release from prison: a nested case-control study." Epidemiol Psychiatr Sci 28(2): 224-233. | do not contein a analysis group of age < 24 years. |
| 36 | Storosum, J. G., et al. (2001). "Suicide risk in placebo-controlled studies of major depression." American Journal of Psychiatry 158(8): 1271-1275. | review of RCT |
| 37 | Su, K. P., et al. (2019). "Comparisons of the risk of medication noncompliance and suicidal behavior among patients with depressive disorders using different monotherapy antidepressants in Taiwan: A nationwide population-based retrospective cohort study." Journal of Affective Disorders 250: 170-177. | do not contein a analysis group of age < 24 years. |
| 38 | Vieweg, W. V., et al. (2006). "Toxicology findings in child and adolescent suicides in virginia: 1987-2003." Primary Care Companion to the Journal of Clinical Psychiatry 8(3): 142-146. | no quantitative analysis of the association of antidepressant use with suicide |
| 39 | Vitiello, B., et al. (2011). "Long-term outcome of adolescent depression initially resistant to selective serotonin reuptake inhibitor treatment: a follow-up study of the TORDIA sample." Journal of Clinical Psychiatry 72(3): 388-396. | no quantitative analysis of the association of antidepressant use with suicide |
| 40 | Wang, M., et al. (2015). "Sick-leave measures, socio-demographic factors and health care as risk indicators for suicidal behavior in patients with depressive disorders--a nationwide prospective cohort study in Sweden." Journal of Affective Disorders 173: 201-210. | do not contein a analysis group of age < 24 years cohort |
| 41 | Weihs, K. L., et al. (2018). "Desvenlafaxine Versus Placebo in a Fluoxetine-Referenced Study of Children and Adolescents with Major Depressive Disorder." Journal of Child and Adolescent Psychopharmacology 28(1): 36-46. | RCT |
| 42 | White, N., et al. (2008). "Suicidal antidepressant overdoses: a comparative analysis by antidepressant type." Journal of Medical Toxicology 4(4): 238-250. | no quantitative analysis of the association of antidepressant use with suicide |
| 43 | Wijlaars, L. P., et al. (2012). "Trends in depression and antidepressant prescribing in children and adolescents: a cohort study in The Health Improvement Network (THIN)." PloS One 7(3): e33181. | The study outcomes were not suicide/suicide attempt/self-harm |

| **Supplementary table 3**. **Study Characteristics of 17 Studies of antidepressant exposure in relation to risk of suicide and suicide attempt** | | | | | | | | | |
| --- | --- | --- | --- | --- | --- | --- | --- | --- | --- |
| **Author** | **Publication year** | **Cohort** | **Country** | **Age at baseline (years)** | **No of participants** | **Mean length of follow-up (years)** | **Study outcome** | **Adjustment** | **NOS (0-9 points)** |
| Valuck | 2004 | Cohort | US | 12-18 | 24119 | 0.5years or more | Suicide attempt (ICD-9/ICD-10) | Propensity for allocation to each treatment group and for demographic and clinical characteristics | 8 |
| Martinez | 2005 | Nested case-control | UK | 10-18 | 21297 | 1 year | Self-harm (medical records) | Adjusted for severity of depression; time depression was diagnosed in relation to start of therapy; referral to psychiatrist or psychologist before index day; history of self harm; diagnosis of, or treatment for, anxiety or panic disorder; schizophrenia; antipsychotic drugs; drug misuse, and alcohol misuse. | 5 |
| Olfson | 2006 | Case-control | US | 6-64 | 3397 | 1 year | Suicide and suicide attempt (ICD-9/ ICD-10) | Sex, age, geographical location (as strata), number of suicide attempts before the index hospitalization, number of suicide attempts during follow-up, use of multiple antidepressant medications, and number of purchased antidepressant prescriptions during the previous year in the model | 5 |
| Sondergard | 2006 | Cohort | Danish | 10-17 | 2311 | 4 years | Suicide (ICD-10) | Age, sex, treatment and psychiatric contact | 7 |
| Tiihonen | 2006 | Cohort | Finland | 10-19 | 15390 | 3.4 years | Suicide and suicide attempt (ICD-10) | Sex, age, geographical location (as strata), number of suicide attempts before the index hospitalization, number of suicide attempts during follow-up, use of multiple antidepressant medications, and number of purchased antidepressant prescriptions during the previous year in the model | 6 |
| Gibbons | 2007 | Cohort | US | 18-25 | 226866 | 0.5 years or more | Suicide attempt (VA health care system) | Adjusted for Differences in Age | 6 |
| Olfson | 2008 | Case-control | US | 6-64 | 1368 | 1 year | Suicide attempt (ICD-9) | Age, sex, race/ethnicity, recent treatment of substance use disorder, recent treatment of depression-related disorder, major depressive episode subtype, symptom severity, and psychotherapy prior to index diagnosis date | 7 |
| Haukka | 2009 | Cohort | Finland | >10 | 57361 | 2.9 years | Suicide (ICD-10) | Sex, age, socio-economic and medical history variables, calendar year, and follow-up time as background variables | 7 |
| Schneeweiss | 2010 | Cohort | British Columbia, Canada | 10-18 | 20906 | 1 year | Suicide attempt (ICD-9) | 500 empirically identified covariates most likely to be confounders as well as the demographic, psychiatric, and clinical covariates | 8 |
| Björkenstam | 2013 | case-crossover | Sweden | >13 | 5 913 | 1 year | Suicide (ICD-10) | / | 7 |
| Cooper | 2014 | Cohort | US | 6-18 | 36 842 | 0.5 years | Suicide Attempts (medical records) | adjusted for medication-speci ﬁ c propensity score and several time-dependent covariates (age, calendar year and month, mental health diagnoses, and use of psycho- tropic medications). | 8 |
| Miller | 2014 | Cohort | US | 10-24 | 102,647 | 1 year | deliberate self-harm (ICD-9) | age | 7 |
| Gibbons | 2015 | Cohort | US | 5-17 | 221028 | 0.5 years | Suicide attempt (ICD-9) | The time dependency of confounders | 6 |
| Christiansen | 2016 | Birth-cohort | Danish | 21 (7-28) | 392458 | 17.7 years | Suicide attempt (ICD-9) | Gender, depression, anxiety and post-traumatic stress, behaviour disorders, personality disorder, type of contact with psychiatric department, use of antipsychotics, contacts to somatic department, parental level of income and parental use of psychopharmacological drugs | 7 |
| Valuck | 2016 | propensity-matched new-user cohort | US | 19-24 | 12673 | 6 months | suicide attempt (ICD-9/ ICD-10) | propensity to receive an SNRI, comorbidities, drug-months of exposure to prior prescriptions and suicide attempt history, among other relevant variables | 8 |
| Linden | 2016 | Cohort | US | 5-18 | 220215 | > 6 months | completed suicide and suicide attempt(ICD-10) | Age, sex, indication of use, antipsychotic use, anxiolytic use and number of different antidepressants prescribed | 8 |
| Joyce | 2018 | cohort | US | <18 | 84 909 | >1 year | suicide attempt (ICD-9) | / | 7 |

# Supplementary Figures.


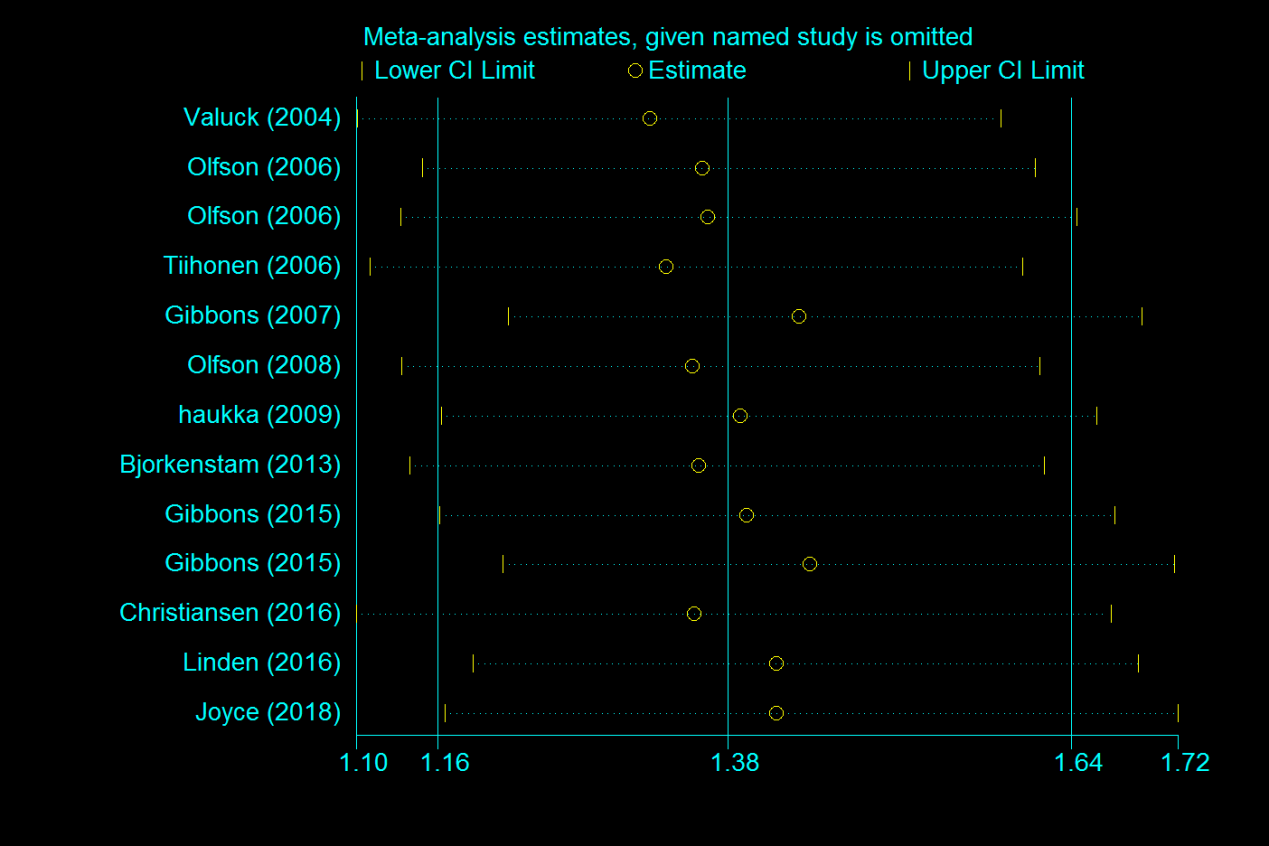


Supplementary figure 1. Sensitivity analysis of antidepressant exposure and the suicidality risk among children and adolescents


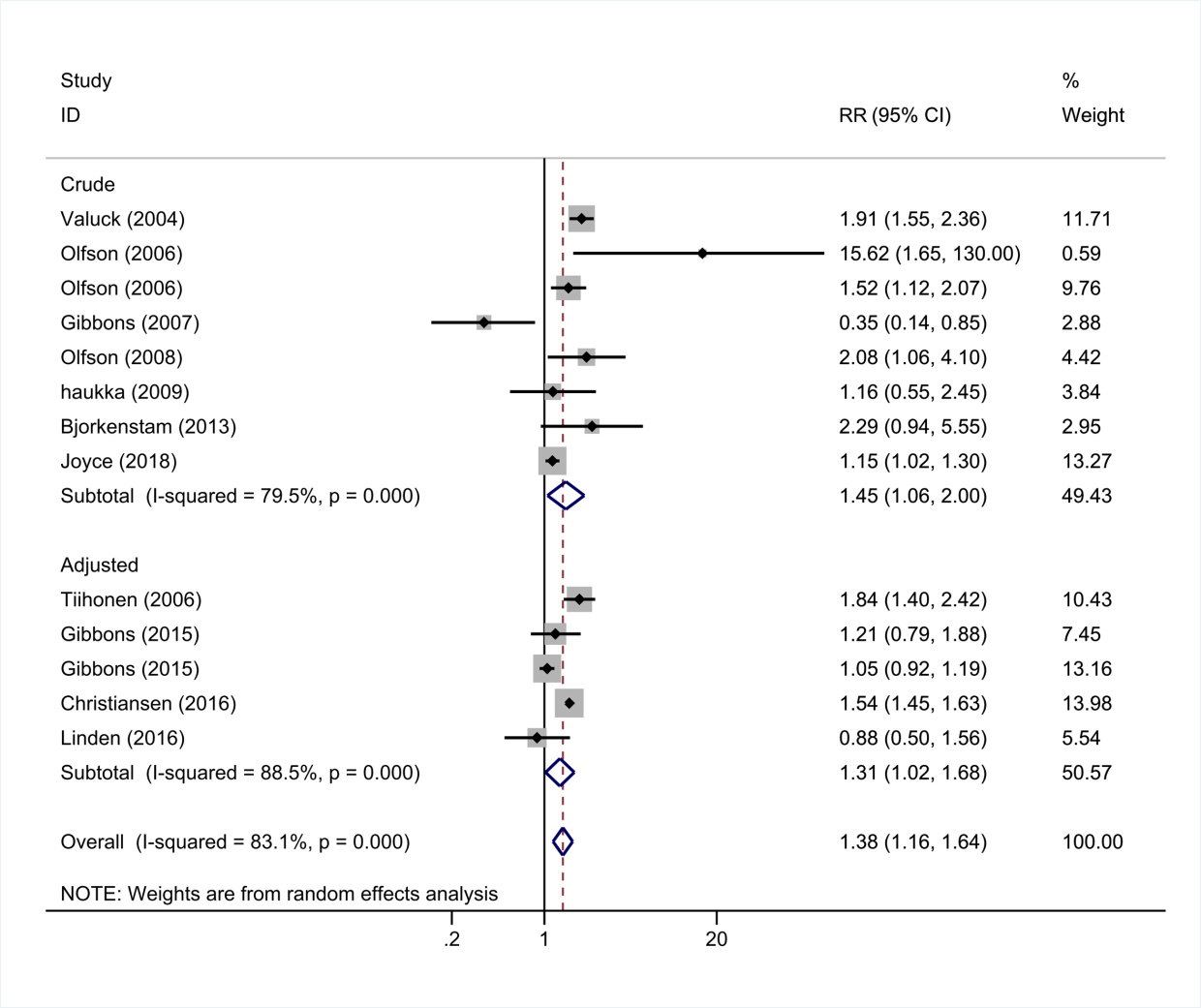


Supplementary figure 2. Subgroup analysis of antidepressant exposure and suicidality risk according to the adjustment_01
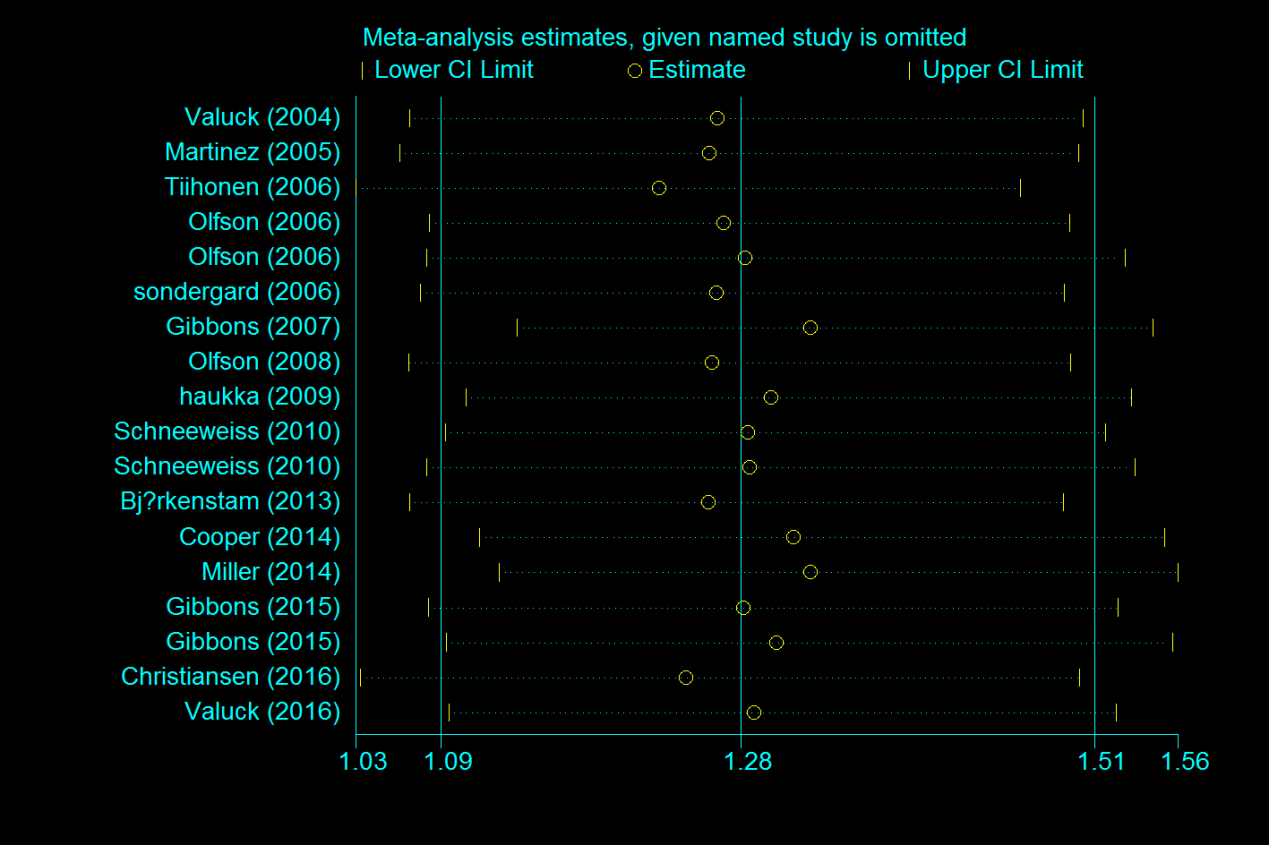
Supplementary figure 3. Sensitivity analysis of SSRI exposure and the suicidality risk among children and adolescents


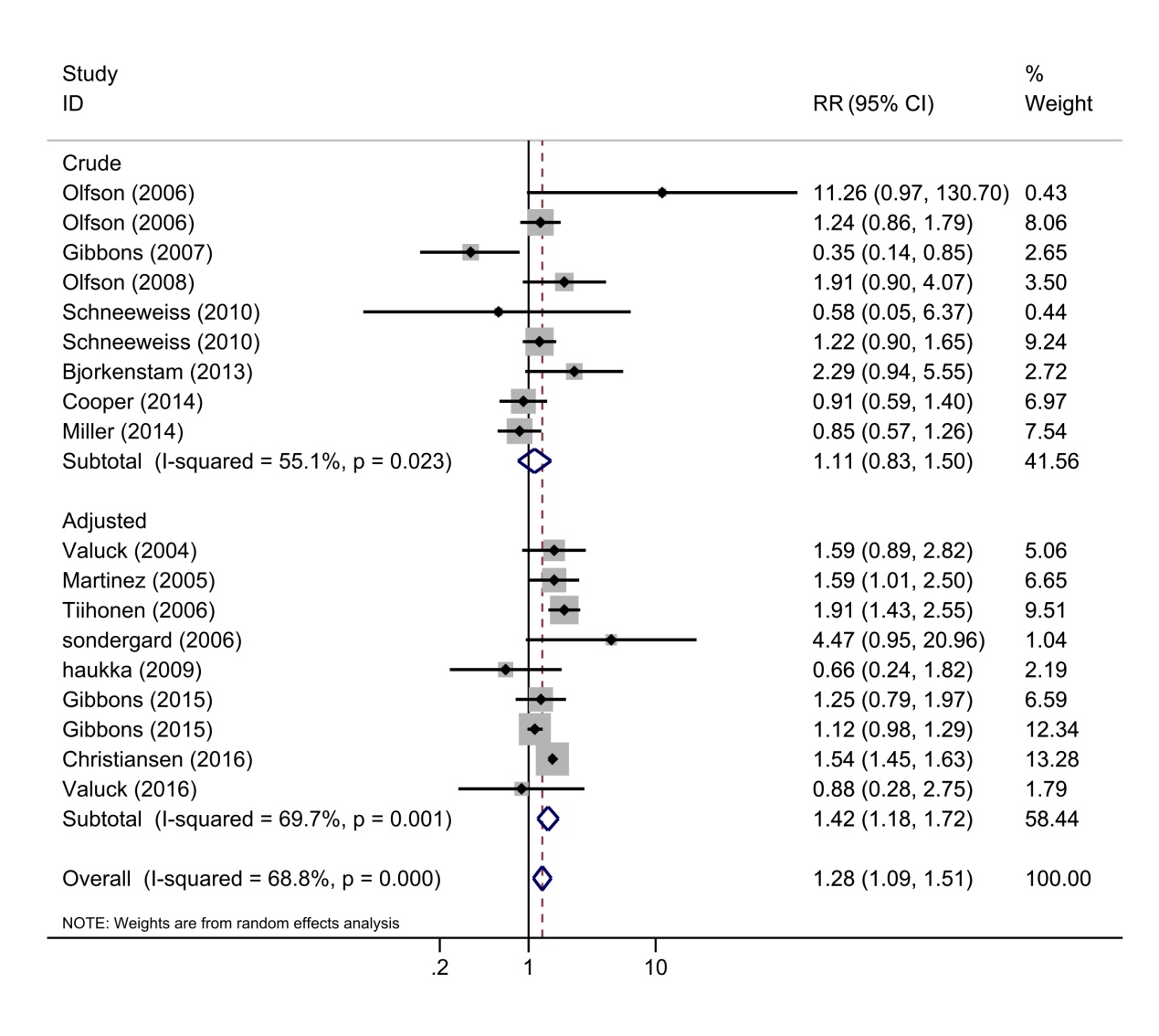


Supplementary figure 4. Subgroup analysis of SSRI exposure and suicidality risk according to the adjustment
